# Science, Scripture, and Sexuality: The US United Methodist Church at Crossroads

**DOI:** 10.1007/s10943-023-01806-7

**Published:** 2023-04-20

**Authors:** Lee Johnson, Barbara Lukert

**Affiliations:** 1 400 W 63rd St, Kansas City, MO 64113 USA; 2 2708 W 50th Terrace, Westwood, KS 66205 USA

**Keywords:** Biology of homosexuality, Scripture, Science, United Methodist Church

## Abstract

During the past 50 years, medical and behavioral scientists have made great progress in understanding the variables which influence the development of sexual orientation, identity, and consequent behavior. In most instances, homosexuality is influenced by hormonal, genetic, and immunologic variables during fetal development, and the effects cannot usually be altered without consequence. The recent struggle within The United Methodist Church in the USA reflects the difficulty that society in general has with accepting homosexuality as part of the spectrum of sexuality. Hopefully, understanding the factors influencing sexual orientation will aid in reducing prejudice and eventually bring an end to the pain endured by the LGBTQ community, and the conflict within The United Methodist Church, a prototype of the struggle.

## Introduction

The purpose of this paper is to examine the conflict within The United Methodist Church (UMC), specifically in the USA, over homosexuality as a micro-cosmos of the struggle of faith communities, and to call attention to the revelations science brings to a more complete understanding of human sexuality. It is our hypothesis scientific evidence pertaining to the spectrum of sexuality can both enlighten and ameliorate homonegativity.

### Evidence that Homonegativity Can be Altered

There is evidence that education about homonegativity can change a person’s attitudes about homosexuality (Baker & Brauner-Otto, 2014; Collier, 2012). A study performed on 125 men and women at a Catholic Liberal Arts University randomly recruited students by posting notices of the study in the Psychology Department. The participants were divided into two groups. One group viewed a video presenting a biological basis to homosexuality suggesting that sexual orientation is predetermined from birth, and the other group viewed a video suggesting that homosexuality is abnormal and urged that individuals should chose to refrain from participating in homosexual behavior. Each participant then took a twenty-item modified Homophobic Scale survey (Lumby, 1976) to assess attitudes toward homosexuals and homosexual orientation. The main outcome confirmed that education about homonegativity can change a person’s attitude toward homosexuality when information presented includes an enhanced understanding of the role of biology (Horton et al., 1993).

### Impact of Diverse SO Beliefs on Homonegativity

A more recent study conducted at the University of Tennessee aimed to investigate the impact of diverse sexual orientation (SO) beliefs on homonegativity (Fry et al., 2022). They studied whether presenting multiple types of SO beliefs could be more effective in reducing homonegative prejudice toward gay men, binegativity toward bisexual men, and infrahumanization toward gay and bisexual men than just focusing on beliefs about biogenetic determinants of SO. They randomly assigned 200 participants (57% men, 78% white) to a treatment or control condition. Participants in a treatment group read one of three essays that summarized research implying that (1) SO is biogenetically determined; (2) SO is socially constructed and countering beliefs regarding the discreteness, homogeneity, and informativeness of SO groups; (3) SO is biogenetically determined as well as research implying SO groups are socially constructed and not particularly discrete, homogenous, or informative.

They postulated participants in both conditions presenting diverse beliefs pertaining to the social construction of SO would report the greatest decreases in negative beliefs in the discreteness, homogeneity, and informativeness of SO groups, in homonegative prejudice, and in binegativity. They expected that only participants in the group reading material excluding biogenetic determinants would report the greatest decreases in infrahumanization, and any observed changes would still be detectable a week after the intervention. They did not observe the results they expected. Only participants in the group discussing biogenetic determinants reported significant decreases in homonegative prejudice and binegativity. There were no changes in infrahumanization. Observed changes were still present a week after the intervention. The investigators suggest it is possible that educational interventions which include biogenetic information when targeting SO beliefs may produce long-lasting reductions in prejudice toward sexual minorities (SMs).

### The Effect of Narrative and Contact on Homonegativity

A study by Kalla et al. sought to determine the effect of sharing narratives to encourage people not in the minority group (ingroup) to engage in perspective-taking, that is, considering outgroup members’ point of view. In this study the minority groups were immigrants and transgender people. They found that 3rd-party narratives (such as those in the case presentations which are included in this paper) were effective in reducing exclusionary attitudes (Kalla et al., 2020).

There is also evidence that contact of heterosexual with homosexual people can decrease prejudice as suggested by the intergroup contact hypothesis. The intergroup contact hypothesis states that contact with members of the outgroup should decrease prejudice against the outgroup (Allport, 1954). A meta-analysis was conducted to examine the relationship between contact and sexual prejudice. A quantitative synthesis from 41 articles, using mostly samples from the USA, showed a significant negative relationship between contact and sexual prejudice, that is, the greater the contact of heterosexual with homosexual people the greater the acceptance of homosexual people (Smith et al., 2009).

A very recent study examined the effect of religiosity on the moderating effect of contact with homosexual people on support of gay rights and same-sex marriage by heterosexual people. They found that contact with gay men and lesbian women can increase the endorsement of specific gay rights and for same-sex unions, but less so among highly religious people (Piumatti & Salvati, 2020).

### The Conflict and Struggle of United Methodists in the USA

In her book *We Shall Not be Moved: Methodists Debate Race, Gender, and Homosexuality,* Nickell suggests the conflict in the UMC over ordination of gays and lesbians and officiating at same-sex marriage is a debate framed as a defense of traditional family values and norms and mirrors the wider secular debate (Nickell, 2014). The struggles of United Methodists in this country over sexual orientation may be instructive for our society as attempts at resolution of the conflict evolve (Nickell, 2014). There is among the populous, a significant lack of awareness of what is known about the biology of the factors influencing the development of sexual orientation. Understanding biological factors will not always change the attitudes of those who are homonegative; however, as noted in the forementioned studies, it can impact attitudes. We believe then, it is important to connect information from both science and religion to make the discussion of human sexuality more complete.

### UMC Doctrine: Homosexuality Incompatible with Christian Teaching

For more than 50 years, The United Methodist Church, the largest protestant main-line denomination in the USA, has held homosexuality as “incompatible with Christian teaching,” standing outside of traditional family values and norms (United Methodist Book of Discipline). Here, it should be noted much of our work draws upon responses of The United Methodist Church located in the USA. Such was the origin of the debate in 1972. However, beginning in 2004, voting at General Conference has been expanded to include an equal representation of world-wide delegates. Every four years since 1972, at its General Conference, the church has renewed this understanding. Pre-pandemic, in February 2019, the church held a specially called General Conference in Saint Louis MO with an expectation the stance would be moderated. However, by a 438–384 vote, with the total consisting of half laity and half clergy, the church re-affirmed its stance and strengthened its punitive language especially toward clergy identifying as “self-avowed and practicing homosexuals.” Following the vote, The United Methodist News Service (Gilbert et al., 2019) reported Bishop Scott Jones, then of the Texas Conference, as saying the vote resolved the long-standing debate about how the church “can best accomplish its mission of making disciples of Jesus Christ for the transformation of the world.” The decision, he said, was consistent with the denomination’s historic stance on human sexuality, as contained in The Book of Discipline since 1972, understood to be the denomination’s authoritative doctrine (Gilbert et al., 2019).

## Scripture, Theological Pluralism, and Homosexuality

This “historic stance” is weighted in what many in the church, bishops, pastors, and laity refer to as the authority of scripture. For United Methodists, theological discourse begins with scripture, meaning it is primary to the discussion, and scripture is to hold authority. For example, when discussing ordination of LGBTQ + persons, the conversation often begins with seven biblical tests, Old and New Testament (Journal of Bible and Culture, April 22, 2015). Hermeneutical study, especially that which employs the tools of historical and cultural criticism, leads some in the church to believe these scriptures cast homosexuality as a sin, outside of God’s law. Others though, using a similar hermeneutical approach, reach a different conclusion pertaining to the authority scripture holds in relationship to homosexuality. Here, the UMC recognizes a theological discussion does not preclude other sources of authority and encourages scholarly inquiry and personal insight to enrich an understanding of the Bible (BOD page 84, paragraph 105).

Such an understanding stems from legislation approved at the same 1972 General Conference which held homosexuality as “incompatible with Christian teaching,” There, at the conference held in Atlanta GA, delegates also approved what the **New York Times** called “landmark doctrinal guidelines, designed to help people understand their religion in contemporary society.” (Blau & NY Times, 1972). The guidelines, called theological pluralism, encouraged United Methodists to measure their beliefs against four criteria, “scripture, church tradition, personal experience, and reason.” The doctrinal statement, approved overwhelmingly, also encouraged varying theological positions within “the framework of basic Christian doctrine.” It was as though the General Conference left open the possibility to further deliberation on a theological perspective and hermeneutical study of homosexuality. To this day, the document remains part of The United Methodist **Book of Discipline** (United Methodist Church, 2016). Here, the idea that scientific evidence, along with personal narrative, can both enlighten and ameliorate homonegativity becomes relevant. Yet, the history of the UMC provides evidence of a tension between the reason of scientific evidence, the narrative of personal experience, and the authority of scripture.

## The Rise of Advocacy Groups: The Good News Movement

The debate at the 2019 General Conference in Saint Louis reflected this tension. It is a tension deeply rooted in United Methodist theological discourse, even predating the decision on theological pluralism and homosexuality. In 1967 the Good News movement formed with a mission to lead “all people within the Methodist Church to faithful and vibrant practice of orthodox Wesleyan Christianity.” Following its 45th anniversary, in a May 2013 article in **The Good News Magazine**, James V. Heidinger II, former president and publisher of the magazine (1981–2009) wrote, “Born (theological pluralism) in an era when church radicals were demanding, ‘Let the world set the agenda for the church,’ we are convinced the biblical agenda was languishing from both neglect and theological revisionism.” Heidinger called theological pluralism a theological malaise writing, “While pluralism may have been included to express some of the legitimate diversity within the parameters of historic Christianity, it was interpreted by many to mean United Methodism offered members a proliferation of theological views, many of which far exceed boundaries of sound biblical practice.” Over the years, citing its hermeneutical understanding of the authority of scripture pertaining to certain biblical texts, the Good News movement has worked to help the church maintain its prohibitive language against homosexuality in **The Book of Discipline.**

In 1992 at the General Conference held in Louisville KY, a study committee recommended removing the prohibitive language from **The Book of Discipline.** The 24-member committee, after spending four years applying the doctrine of theological pluralism to its work, consulting “recognized scholars in the fields of biblical interpretation, theology, psychology, medicine and sociology,” concluded “the church cannot responsibly maintain the condemnation of all homosexual practice.” (From “The Church Studies Homosexuality. 1994. Nashville, Tenn: Cokesbury). However, the tension between theological pluralism and the authority of scripture surfaced when the committee’s minority report was adopted by the conference. It stated, “The present state of knowledge and insight in the biblical, theological, ethical, biological, psychological, and sociological fields does not provide a satisfactory basis upon which the church can responsibly alter its previously held position that the practice of homosexuality is incompatible with Christian teaching.” The decision was weighed in the authority of scripture, excluding the insights of, most noticeably, personal experience, and reason.

## Other Voices of Advocacy

Others within the denomination, both groups and individuals, have sought to understand scripture’s authority alongside personal experience and reason, inclusive of hermeneutical study. For these groups and people, full inclusion of LBGTQ + persons does not exceed “boundaries of sound biblical practice.” In the early 1980s, Affirmation Groups, largely made up of persons from the gay and lesbian communities, formed throughout the denomination. The groups advocated for full inclusion. In 1982, an Affirmation Group in Boston MA developed an idea to help local churches and individuals declare support for full inclusion of gays and lesbians within the church structure. The meeting inspired the name “Reconciling Congregations.” In May of 1984, following a vote by The United Methodist General Conference that further strengthened the **Book of Discipline’s** prohibitive language, banning “self-avowed and practicing homosexuals” from ordination, a group of 12 Affirmation members distributed material to General Conference attendees inviting churches to become Reconciling Congregations and dissent from the **Book of Discipline**. Today, more than 40,000 United Methodists, laity and clergy, are members of the Reconciling Network.

### Individual Dissent: Jimmy Creech and Frank Schaefer

Notable individuals who dissented from **The Book of Discipline** included Rev. Jimmy Creech and Rev. Frank Schaefer. In September of 1997, Creech, serving First United Methodist Church in Omaha NE, officiated a union between two women. Charges were filed for violation of **The Book of Discipline.** In March of 1998, Creech was acquitted. However, Nebraska Annual Conference Bishop Noel Martinez did not allow him to continue at First UMC. In April 1999, Creech officiated a union between two men while in Chapel Hill NC. He was again brought to trial in Nebraska and refused to enter a plea stating, “Doing so would legitimize church law.” During the trial, Creech addressed hermeneutical study of the scripture generally used to condemn homosexuality saying to the jury, “Some give evidence of Sodom and Gomorrah, Leviticus, Romans and a few other passages, which have an allusion to same sex relationships.” Some also fear compromising on these passages would allow biblical authority to be eroded. “Yet,” said Creech, “they are only protecting one interpretation of scripture.” This time, Creech was found guilty.

In 2007, Schaefer, pastor of the Zion United Methodist Church of Iona in Lebanon PA., officiated a marriage between his son and partner in Boston MA where same-sex marriage was legal. In 2014, Schaefer was brought to trial and found guilty. A church appellate court overturned the verdict because seven years had passed between the marriage and charges. In an interview with Zoraida Sambolin of CNN, Schaefer said, “(He) once believed homosexuality was incompatible with his Christian beliefs, but his views changed when his son came out.” Schaefer’s experience affirms the previously noted intergroup contact hypothesis.

### The Exclusion of Science

This tension between the authority of scripture and theological pluralism, both doctrinal standards within The United Methodist Church, has been persistent. A February 1, 2021, article from **The Good News Magazine** reflects the persistency. In it, Rev. Thomas Lambrecht, Vice President, and General Manager of Good News, reaffirms the authority of scripture in relationship to understanding homosexuality. Rev. Adam Hamilton, United Methodist Church of the Resurrection Senior Pastor in Leawood, KS, the denominations largest church, responded to Lambrecht. Hamilton’s hermeneutical study, especially that which is grounded in historical and cultural criticism, does not consider all scripture to hold equal authority and has said such in previous books, “*Making Sense of the Bible”* (Hamilton, 2014) and “*When Christians Get it Wrong*” (Hamilton, 2013). In his work, Hamilton encouraged the church to become more welcoming to the LGBTQ + community. The back-and-forth nature of the Lambrecht/Hamilton dialog reflects well upon a discussion persistently mired in the tension between scriptural authority and theological pluralism in relationship to human sexuality.

It should be noted, these discussions occur at a time when science has helped stem the COVID-19 pandemic. Almost, without exception, the discoveries of science in relationship to COVID-19 are celebrated, even in the church. Yet, in The United Methodist Church it appears there is a disconnect with scientific discovery when it comes to a more complete understanding of human sexuality. This disconnect, and the tension between the authority of scripture and theological pluralism, is reflected in another paragraph of **The Book of Discipline** which says science, while a “legitimate interpretation of God’s natural world,” is precluded from “making authoritative claims about theological issues.” (BOD, paragraph 160 F). Here, **The Book of Discipline** understands homosexuality from a theological perspective; thus, the authority of scripture ignores the evidence of science. In their back-and-forth dialog in 2021, neither Lambrecht nor Hamilton inserted science into the conversation.

### The Crossroad of Schism

A year later, in March of 2022, the Global Methodist Church formed, creating the first schism within The United Methodist Church since its formation in 1968. Driven by the on-going discussion within the church in relationship to LGBTQ + ordination and marriage, Rev. Keith Boyette, chairman of the Global Methodist Church’s transitional leadership council said, “Theologically conservative local churches and annual conferences want to be free of divisive and destructive debates and have the freedom to move forward together. We are confident many existing (United Methodist) congregations will join the new Global Methodist Church in waves over the next few years.” There appears to be some truth to Boyette’s prediction. By October of 2022, the United Methodist News reported nearly 500 United Methodist Churches in Texas, including 4 of the 6 largest, have disaffiliated. At the same time, The United Methodist Church announced plans to postpone its General Conference for 2022, scheduling it in 2024 when the church will continue its discussion of human sexuality.

### Connecting Theology and Doctrine with Science

It is here, returning to our hypothesis, we propose church leaders within The United Methodist Church, both laity and clergy, work to help members connect theology, science, and doctrine in its understanding of human sexuality. The conflict created by this 50-year discussion, and the more recent schism, illustrates the dangers of ignoring a rational, evidence-based understanding of human sexuality. There is ample evidence of hermeneutical study from theologians and scholars on both sides of the conversation pertaining to homosexuality (DeLong, 2000; Gomes, 2002; Kimball, 2008; Roberts et al., 2007; Gnuse, 2015). It has done little to move forward the discussion. Our hypothesis and work here is that the conversation will move forward by including scientific evidence.

## The Science of Sexual Orientation

In spite of holding very definite opinions on the acceptance of persons in the sexual minority, many church hierarchies and lay governing bodies have ignored discoveries in the world of science which inform our understanding of the biology of sexual orientation and identity. Scientists and religious leaders have been negligent in making this information readily available. As a result, the church continues to see sexual variations as a moral issue, informed by scripture, and used to defend “traditional family values and norms.” In this article we are attempting to contribute a non-scientist-friendly overview of the body of scientific evidence that informs our understanding of the biologic influences on sexual orientation. We propose that this is an important element in reframing the discussion from one considering only moral aspects of sexuality to a discussion which includes a scientific view of sexuality.

It was an understanding of scientific and sociological studies that led the American Psychiatric Association in 1972 to remove homosexuality from its list of mental disorders (1974*Diagnostic and statistical manual of mental disorders*). In 1948, Dr. Kinsey reported the results of a large study of sexuality introducing the concept of a spectrum of sexuality ranging from individuals who have no attraction to others of the same sex, to people who are sexually attracted to both sexes, to individuals who are attracted only to people of the same sex. This finding highlighted the realization that there are multiple factors that influence the development of various forms of sexual attraction, and there is significant variability in its fluidity (Kinsey, 1945).

### Biologic Factors Influencing the Development of Sexual Orientation

More recently, scientists have discovered the factors contributing to the development of sexual orientation include hormonal effects on the sexually dimorphic nucleus of the preoptic area of the brain (SDN-POA) during fetal development, effects of specific genes and mutations of genes, immunological factors in the mother, the interaction of molecular and environmental factors, and interactions between genes and hormones (Balthazart, 2011).

The effects of hormones on the prenatal brain appear to have the greatest influence on the development of sexual orientation (Bao & Swapp, 2011). The fetal gonads, the major source of sex hormones, develop in early pregnancy, while the brain develops during the second half of pregnancy. Sex hormones, estrogen, testosterone, and progesterone, act on the brain by binding to receptors on the brain cells. Once the hormone enters the cells it initiates the production of neurotransmitters, chemicals which control behavior. The effect of sex hormones may be modified by differences in the receptors in the brain cells. The quantity of hormone entering the cell is the dominate controlling factor. The anatomical distribution in the brain at sites of action of sex hormones determines the effects of sex hormones on human behaviors such as sexual orientation.

Evidence for the effects of hormones on the SDN-POA area of the brain is derived from two sources: observations in nature (naturally occurring variations in both animals and humans) and experiments in animals.

### Observations in Animals

A spontaneous model of homosexuality has been described in sheep in the Northwest section of the USA (Roselli et al., 2007, 2020). When 700 rams were offered a choice between a male or female partner, 51% exclusively chose a female, 31% were bisexual, 10% were asexual, and 8% were exclusively male oriented (homosexual). The SDN of the male-oriented (homosexual) sheep were significantly smaller and contained fewer neurons than in the males oriented to females (heterosexual), making them more similar to females. The aromatase activity was also lower in the preoptic area in male-oriented males (Balthazart, 2011).

Same-sex behavior has been described in Japanese Macaques (Vasey et al., 2014). Many of these primates choose an enduring female partner even when given a choice to be with a male alternative. According to some reports, approximately 1500 species of animals have been observed to engage in same-sex sexual relationships (Kamath et al., 2010). We have not searched all of those described to determine whether all choose a same-sex partner even when offered an opposite-sex alternative or whether the relationship is life long. Although observations in animals cannot always be applied directly to the human condition, they do allow the study of hormonal and genetic factors that for ethical reasons cannot be performed in humans.

### Animal Experiments

Animal studies show that sexual attraction to same or opposite sex is modulated by the SDN-POA, which is programmed by prenatal hormones. Studies have shown the number of cells in SDN-POA of males is 5–6 times larger than in females due to action of testosterone during embryonic development and/or during the first days after birth. This effect is irreversible (Bao & Swaab al., 2011; Roselli et al., 2007). Absence or low concentrations of testosterone will lead to a female pattern in the male fetus, i.e., attraction to male partners. Testosterone is converted to estrogen by aromatase activity in the cell; therefore, estrogen is actually the final signal. We will refer to the hormone that initiates the cascade of responses, and this hormone is usually testosterone with its action requiring conversion to estrogen. If rats are given aromatase inhibitors (which prevent testosterone from being converted to estrogen), during the end of embryonic life or the first week after birth, the male rats develop into adults that are attracted to other males. Homosexual orientation induced by perinatal hormone treatment cannot be modified by hormones given in adulthood.

### Observations in Humans: Patient One

Observations in humans confirm the aforementioned animal experiments. A representative case in point illustrates a perspective-changing narrative: A patient consulted an endocrinologist because she was concerned about being attracted to girls.^1^ In early childhood she preferred activities more typical of boys. She had delayed onset of menstrual periods and was treated with hormones. She noticed increased facial hair at about age 12 years. She began to be sexually attracted to a girl at about age 12 years, and at age 19 she developed a romantic and physical relationship with a sorority sister. She was troubled because her church and family considered this a sin. She had tried to end the relationship but was suffering great emotional pain. Her question to the endocrinologist was whether she had abnormal hormones which were causing this problem. An endocrine evaluation revealed that the patient had congenital adrenal hyperplasia (CAH), an enzyme variant in her adrenal gland which caused her to shunt hormone production to testosterone formation resulting in elevated serum testosterone levels.

In this syndrome the enzyme variant is present during fetal development. Girls exposed to high testosterone concentrations before birth due to an enzyme variant (e.g., congenital adrenal hyperplasia in this patient) exhibit increased aggressiveness and greater interest in male-typical activities. In a 1996 study, Zucker found that ~ 40% of girls with congenital adrenal hyperplasia experience some form of homosexual attraction (Pasterski et al., 2014; Zucker et al., 1996). In a review of observational studies on the sexual orientation of patients with CAH published between 1985 and 2016, Gondim concluded that the prevalence of homosexuality and bisexuality is greater in patients with CAH compared to the general population (Gondim et al., 2018). The rates of bisexual and homosexual orientation were increased above controls not only in women with classical CAH, but also women with “non-classical” form, and correlated with the degree of prenatal androgenization, supporting the theory that the elevated concentrations of testosterone during fetal development induce the sexually dimorphic nucleus of the supra optic area of the brain to be programmed for a sexual orientation typical of a male fetus, that is, attraction to women (Meyer-Bahlburg et al., 2008).

It is not surprising that fewer than100% of the female patients with CAH developed female sexual preference. Differences in timing of the elevated levels of testosterone, other downstream factors influencing the response to higher testosterone concentrations, postnatal social and environmental factors are all possible variables affecting behavioral responses to the effects of testosterone.

### Observations in Humans: Patient Two

There also are important interactions between genes and hormones, as illustrated by the following case prototype, another example of a potentially perspective-changing narrative: A 23-year-old woman consulted an endocrinologist with the complaint, “I’ve never had a menstrual period.” At age 10 years she began to develop breasts and had normal female characteristics. She recently became engaged and decided that she should have an evaluation regarding her absent menses. Endocrine evaluation revealed that the patient had an XY (male) chromosome pattern and high blood levels of testosterone. She had intra-abdominal testes. A uterus was not present. After removal of the testes and construction of a vagina, the patient was happily married for over 40 years. She and her husband adopted 2 children.

The presence of XY chromosomes indicates that genetically this patient is male. The Y chromosome codes for the development of male sex in the fetus. Males have an X and a Y chromosome, while females have two X chromosomes. Patients such as the one described have mutations in the receptor for testosterone; hence, testosterone cannot enter their cells to exert its effect (Hamer et al., 1993). XY babies (males) with complete insensitivity to testosterone are born with female genitalia and frequently raised as girls. This is evidence that hormonal effects on the brain during development prevail. Even though genetically a male, the absence of a testosterone effect on the prenatal brain due to abnormal receptors for testosterone, results in female sexual orientation, that is, the individual is attracted to males (Bakker, 2018; Wisniewski, 2000). Similar effects can be produced by mutated enzymes which affect formation or metabolic activation of testosterone. In women with total testosterone resistance, the presence of heterosexual, female sexual orientation (attraction to men), and feminine gender traits, in spite of having XY chromosomes, is consistent with the idea that the action of testosterone is the key biological factor in the development of sexual orientation and gender identity. It does not rule out socialization playing a role since these individuals look like and are raised as females.

One could ponder the question: Should this person whose genes would say they are male but whose physical appearance is female, who is sexually attracted to men, be labeled as heterosexual or homosexual? We do not mean to imply that cases such as the two presented here are common causes of homosexuality. However, study of such cases helps us understand the molecular mechanisms involved in determining sexual orientation.

Scientists continue to identify the multiple factors which influence the expression of genes. Although sex is determined by genes, sexual differentiation of non-gonadal tissue is controlled by gonadal hormones. Alterations, such as methylation of DNA or of histones, can alter the expression of a gene. There is much remaining to be learned about the regulation of these processes (Arnold, 2020; Forger, 2018).

### The Role of Genetics

The pattern of homosexuality in families suggests that genetics plays a role. If a boy is gay, there is a 20–25% likelihood his brothers will be gay. Similarly, lesbian women have a greater probability of having a lesbian sister. Sexual orientation tends to be transmitted through the matriarchal lineage: A gay man has a higher probability of having gay uncles and cousins on his mother’s side of the family (Hamer et al., 1993; Currin et al., 2015). In spite of these patterns, it is difficult to identify specific genes associated with homosexuality. It has proved difficult to identify single genes associated with any of the behavioral traits, even those known to be highly heritable. A number of studies have found the region Xq28 on the long arm of the X chromosome to be influencing male sexual orientation (Hamer et al., 1993; Sanders, 2015; Drabant, 2012). Several other studies of this area showed negative or weak associations (Hu et al., 1995; Mustanski et al., 2005; Ramagopalan et al., 2010; Rice, 1999). The largest (*N* = 477,522) genome-wide-association study of same-gender sexuality was published in 2019 (Ganna et al., 2019).

Multiple genes were found to be significantly associated with ever engaging in same-gender sexual behavior, accounting for between 8 and 25% of variance in this outcome. The investigators state that the findings do not allow meaningful prediction of an individual’s sexual behavior, and associations involve multiple genes in the same person rather than a single gene. Advances in DNA sequencing and the ability to sequence the entire genome of gay populations may facilitate the quest for mutations associated with homosexuality. However, in reviewing this study, Diamond points out the multiple factors complicating interpretation of the association between genes and sexual behavior, particularly in differences between sexual attraction and sexual behavior and how that is interpreted by participants in the study, and even differences in what people consider to be a sexual relationship, and whether the relationship is short term or enduring, and the powerful influence of the culture in which the people live that molds their behavior. Those interested in a more intensive discussion of these issues are referred to Diamond’s review (Diamond, 2021).

Determining the magnitude of the effect of genetics on the development of sexual orientation is difficult. There is marked variation in the results of such studies. Studying concordance of effects of genes in identical twins would seem to be the ideal model. One study reported that if an identical twin is gay, there is 65% chance his twin brother will be gay (Whitam et al., 1993). More recent studies show heritability varying from 50% in both sexes (Bailey & Pillard, 1991, 1993) to 30% for men and 50- 60% for women (Kirk, 2000), to 0% for men and 48% for women (Hershberger, 1997), with very different heritability in different populations (Alanko et al., 2009; Kendler et al., 2000; Kirk et al., 2000). These wide variations in heritability are not surprising. Recent studies have shown that there are genetic differences even in identical (monozygotic) twins. A study of 387 identical twin sets in Iceland found up to 100 genetic differences per set of identical twins (Jonsson, 2021). Even with identical genes the expression of a gene can be influenced by hormonal and/or environmental factors, and mutations can occur in only one twin, causing changes in exposure to hormones or structural changes in the brain in utero. Thus one would not expect perfect concordance of characteristics, even in identical twins, as manifest also in differences in finger prints, height, skin tone, and/or birthmarks. The conclusion after reviewing the multiple studies is that genes are among the factors that influence the development of sexual orientation. Differences in heritability can differ between populations in the absence of differences in the associated genes. These differences are presumably due to variations in the strength of the non-genetic effects increasing or decreasing the effect of genes on the trait being studied, in this case, sexual orientation.

### Immunological Effect

It is also likely that immunological response affects the development of sexual orientation during gestation. In any family the second-born son is 33 percent more likely than the first to be gay, and the third is 33 percent more likely than the second. It is postulated that the mother develops an immune response to hormones or other antigens; e.g., a Y-linked protein produced by each new male fetus (Bogaert et al., 2017). This could lower the testosterone available for its effect on the developing brain resulting in female sexual orientation, that is, attraction to males (Bogaert & Skorska, 2011; Bogaert et al., 2017). For an excellent discussion of this phenomena, beyond the scope of this discussion, see (Balthazart, 2017).

## Summary of Biologic and Genetic Influences

In summary, the development of sexual orientation is affected by a wide array of factors. The overriding biologic determining factor appears to be hormonal. Genetic and hormonal control overlap. For example, the higher incidence of homosexuality in patients with congenital adrenal hyperplasia is most likely due to the exposure to high concentrations of testosterone during fetal development. The elevated testosterone is the result of a mutation in the CYP21A2 gene. Because of the complexity of contributing biologic factors and their interaction with social/environmental factors, not all individuals with any one of the variations in genetic, hormonal, or environmental influences will manifest behavior related to same-sex orientation; however, when there is a predominance of same-sex behavior in, for example people exposed to high levels of testosterone in utero, it is reasonable to assume a relationship of the behavior to the hormonal level.

### Fluidity of Sexual Orientation

There will always be differences of opinion concerning the fluidity of sexual attraction and behavior. The tendency of investigators to classify people as either homosexual or heterosexual, rather than recognizing the spectrum of sexuality, has greatly complicated the study of fluidity. In designing studies it is also important to distinguish between physical attraction; i.e., the desire to engage in actual sexual contact, and romantic or emotional attraction, a desire for intimacy that is not necessarily expressed as sexual contact. The development of sexual orientation is strongly influenced by biologic and hormonal factors, while sexual behavior is significantly affected by social and environmental milieu. In many cases the attraction may not change, but the engagement in sexual relationships may be prohibited by social pressure and the desire for a more conventional relationship (Katz & Hyde, 2017).

The data regarding the prevalence of sexual fluidity in people identifying as homosexual vary from 9.5 to 26–45% for men (Mock & Eibach, 2012; Katz-Wise & Hyde, 2015). More recent information relying on prospective studies may yield more reliable results. In one of the early prospective studies Diamond interviewed 89 non-heterosexual young women. When interviewed 5 years later 81 of them were still non-heterosexual. Heterosexual women were not included in the study. Interestingly, there were more changes in the labels that the women applied to themselves than in the direction of their attractions (Diamond, 2003).

There are few large prospective studies of the change or stability of sexual orientation over time. Most early studies that have been reported are retrospective. Two prospective studies reported widely differing findings of the prevalence of fluidity. A 10-year survey of 2500 men and women were studied in the National Survey of Middle Life Development in the USA (O). In this study, 97.4% were heterosexual, 1.33% bisexual, and 1.25% homosexual. Two of 21 (9.5%) of men who identified as homosexual experienced a change in sexual attraction, one to bisexual and one to heterosexual. Bisexual men, bisexual women, and homosexual women showed greater prevalence of change: 47%. 65%, and 64%, respectively, reported a change in sexual orientation (Mock & Eibach, 2011). Note that even though the percentages seem high, the actual numbers studied were very small (21 men). Obviously, very large numbers of people must be surveyed in order to identify adequate numbers of non-heterosexual subjects for a meaningful study. The lack of larger prospective studies hampers our ability to estimate the probability of change of sexual orientation. Change does occur; however, it is clear that sexual orientation seldom changes in heterosexuals, occurring in 1.4% of women and 0.8% of men in the Mock study (Mock & Eibach, 2011).

### Factors Contributing to Fluidity

Factors contributing to sexual fluidity are numerous. Scheitle utilized a representative panel of US adults (*N* = 1034) surveyed in 2010, 2012, and 2014 by the General Social Survey (gss.norc.org) to examine the fluidity of sexual identity. They found that 2.40% of US adults reported at least one change in sexual identity across the 4 years, with 1.59% reporting one change and 0.81% reporting two changes. They found that lesbian or gay individuals (*N* = 17), bisexuals (*N* = 15), and females (*N* = 585) showed more sexual identity fluidity compared to heterosexuals (*N* = 1003) and males (*N* = 450), respectively. Marital status, age, race, and education did not have significant associations with sexual identity fluidity. They also examined the role of religion, to determine whether religion can contribute to destabilizing sexual identity and prolong the development of sexual identity. Higher levels of religiosity make it more likely that lesbian or gay individuals will be fluid in sexual identity, but this is not the case for heterosexual individuals. This finding is in agreement with past qualitative research that has suggested that religion can extend or complicate identity development (Scheitle & Wolf, 2017). Some believe that considering sexual orientation immutable implies that same-sex attraction is inferior to other sexual attractions (Diamond & Rosky, 2016; Rosik et al., 2021)*.* Unfortunately, some authorities who believe that sexual orientation is fluid in most individuals, reject the observations indicating that genetic and hormonal factors play an important role in development of sexual orientation. We believe that the human and animal data we have reviewed in this paper provide valid evidence of the important influence of genetics and hormones. This does not need to be an either/or debate. We have emphasized the biological components that influence the development of sexual orientation because we feel that this information is too often ignored even though the scientific evidence has been validated in multiple laboratories, and has been peer reviewed and published as referenced throughout this paper. Whether or not a person’s sexual orientation can change, probably depends on the strength of the biological and environmental influences versus social pressure. For individuals surrounded by a powerful social majority which proclaims that the practice of homosexuality is a mortal sin, it is most comfortable to live a heterosexual lifestyle. In many cases the resulting constant conflict takes an emotional toll which is manifest in other behaviors such as anger, anxiety, and chronic depression.

After analyzing sexual fluidity in a study in the Netherlands, Diamond and Rosky summarized their observation: “the data on change are relatively clear: Although therapeutic attempts to change sexual orientation are not successful, patterns of same-sex and other-sex attractions sometimes change on their own, and the overall social climate of viability and acceptance regarding same-sex sexuality may be one of the factors influencing such change.”

It is important to remember that homosexuality is not simply a different sexual orientation; it is also associated with complex physical, behavioral, and functional changes ranging from gender-specific differences in play habits as children, to cognitive differences such as visuospatial traits, differences in the size of the dimorphic cell group in the hypothalamus, differences in finger length and ratio of the length of arms to trunk length (LeVay, 2017).

### Science, Society, and the Church

In 2019 the Pew Research Center conducted a global survey on the acceptance of homosexuality. They report that acceptance is influenced by multiple factors, one of which is the level of education. In the USA, 79% of people with post-secondary education believed that homosexuality should be accepted, while only 66% of those with less education agreed. This difference is even greater in some countries, e.g., in Bulgaria 55% of those with post-secondary education favor acceptance of homosexuality compared to 25% of less educated individuals. It is likely that those with higher education are exposed to a greater extent to the scientific views of sexual orientation, i.e., the factors influencing the formation of orientation (Poushter & Kent, 2020).

### The Scientific Attitude

McIntyre in his book “The Scientific Attitude*”* presents the belief that what is distinctive about science is the scientific attitude toward empirical evidence. He says, “To do science we must be willing to embrace a mindset that tells us that our prior beliefs, ideologies, and wishes do not matter in deciding what can pass the test of comparison with the evidence…. At its heart, what is distinctive about science is that it cares about evidence and is willing to change its theories on the basis of evidence.” (McIntyre, & Lee, 2019) Given this approach, the results of investigations of scientists should be valuable in reassessing the views of society toward homosexuality. Turner emphasized in this journal the importance of faith leaders understanding the science of sexuality when he said, “Faith and theology cannot ignore the science of sexuality any more than it can ignore the science of evolution.” (Turner et al., 2012) Conversely, for those who believe that “science has nothing to offer that would even remotely constitute persuasive evidence that would compel us to deviate from the historic Christian judgment that full homosexual intimacy, homosexual behavior, is immoral….. How can science have any relevance for a religious position?” (Jones & Yarhous, 2000). For those who embrace this philosophy, an understanding of the biologic causes of homosexuality has no influence on how they judge behavior. Thus we propose, science cannot determine morality, but it can expand the understanding of human behavior which may encourage grace when judging behavior.

Haidt in his book *The Righteous Mind* suggests that beliefs, particularly those related to morality, are emotional, not logical (Haidt, 2012). As a result conservatives and liberals lack a common language and have different understandings of right and wrong. These are genuine differences and we are all products of the culture in which we were born and/or live. It seems to us moral judgments have a tendency to evolve from cultural experience rather than reason. Because of this, it is our hope that by applying perspective-getting narrative by sharing the stories like those of the patients presented here creates a better understanding of and empathy for those with minority sexual orientation.

### Effect of Religion on the Mental Health of Non-heterosexual People

Meladze discussed the unhealthy role that religion can play in promoting homonegativity (Meladze & Brown, 2015). Meladze studied the ability of gay men who follow one of the three Abrahamic religions (Christianity, Islam, or Judaism) to resolve the conflict between their homosexuality and their religion. 50% of gay men were unable to resolve this conflict. The resulting cognitive dissonance is associated with internalized homonegativity directed toward themselves as well as toward homosexuality in general. Individuals who cannot resolve this conflict develop internalized shame, low self-esteem, higher risk for having eating disorders, isolation and problems with relationships. This is less likely to occur in gay men with no religion or those with Philosophical/New Age religious beliefs, religions which do not condemn homosexuality (Meladze & Brown, 2015).

Evidence from healthcare providers further reveals the harm inflicted on the health of sexual minority youth. Surveys by the Center for Disease Control (CDC, 2021) revealed significantly higher risk for negative health outcomes for lesbian, gay, and bisexual, known as sexual minority youth (SMY) than for heterosexual students. SMY are about twice as likely to be bullied, use illicit drugs, feel persistently sad or hopeless, and 3 × as likely to inject illegal drugs and consider suicide and more than 4 × as likely to attempt suicide (Gibbs & Goldbach, 2015) . The most likely reason LGBTQ youth and adults are at higher risk is minority stress, i.e., daily exposure to stigma and discrimination. Rejection by family and faith communities contributes significantly to this stress (CDC, 2021).

National large-scale studies collected by the University of Texas at Austin’s Research Consortium, produced information about the mental health of college students (Blosnich et al., 2020; Lytle et al., 2018). In a study completed in 2011, the consortium analyzed data from 21,247 college students, age 18–30. Respondents were asked to rate how important their religious or spiritual beliefs were to developing their personal identity. Students were also asked a number of questions about whether they had ever seriously considered or attempted suicide. (We quote here from that report.) The study found that religion may have acted as a protective factor against suicide attempts among heterosexual youth. Each increase in the level of importance of religion among straight youth was associated with a 17 percent reduction in recent suicide attempts. In contrast, for lesbian and gay youth, increasing levels of religious importance were associated with increased likelihood of recent suicidal ideation. Lesbian and gay youth who said that religion was important to them were 38 percent more likely to have had recent suicidal thoughts, compared to lesbian and gay youth who reported religion was less important. Among lesbians, religiosity was linked to a 52 percent increased chance of recent suicidal ideation. Questioning youth whose religion was important to them were nearly three times as likely to have attempted suicide recently, compared to questioning youth who reported religion was less important. The importance of religion was not significantly associated with suicidal ideation or suicide attempts in bisexual individuals. Transgender individuals are also at increased risk for suicide, but the number of transgender people in the study of the relationship to religiosity was not sufficient for analysis (CDC). “These findings should be very sobering and thought provoking for religious leaders as they consider how they care for LGBTQ youth.” (Blosnich et al., 2020). A recent study by Oh noted that the importance of religion was significantly associated with lower odds of suicidal behaviors for heterosexual students when compared with sexual minority students (Oh et al., 2022). A smaller study by Rosik et al. found that several markers of “religiousness” were not directly associated with either improved or worsened health outcomes for depression or anxiety. However, religious activity moderated the influence of internalized homonegativity (IH) on depression such that IH was less strongly related to depression among individuals who frequently attended religious services than among individuals who infrequently attended religious services (Rosik et al., 2022). This suggests that regardless of the official stance of a denomination regarding sexuality, an LGBTQ individual may find solace in the worship experience shared with the congregation.

## Limitations

We did not adequately address information pertaining to transgender people except to recognize their plight as a sexual minority. Neither did we discuss the stress on families and friends of LGBTQ + persons resulting from homonegativity in their communities.

We did not explore the effect of sexual orientation minorities on society, exactly what characteristics of sexual minorities make non-minorities feel uncomfortable, nor how a better understanding of sexual orientation influences the attitudes of society toward persons of the minority group. Our hypothesis holds that understanding science, and the biology which strongly influences the development of sexual identity, can result in a better understanding of human sexuality, and ease the conflict in the church, especially The United Methodist Church. To test this hypothesis we propose a study designed to investigate the effect of including science in education aimed at increasing the understanding of differences in sexual orientation to decrease homonegativity among clergy and lay leaders in The United Methodist Church. If successful, eventually extending this type of education to all laity and clergy may diminish the pain of division caused by differences in attitudes toward sexual minorities.

The authors do not suppose the hypothesis is values free. It reflects the contextual histories of the authors, one a United Methodist lay person and Professor Emerita in the Division of Endocrinology, Metabolism & Genetics in the Department of Medicine in the University of Kansas Health Systems; the other an ordained United Methodist Pastor with advanced degrees in education and divinity, having served in local churches and in a United Methodist Seminary since 1984. The authors portend an ecclesiological understanding of the church which they believe is evidenced in Pentecost where the Holy Spirit brought together diverse believers to form the church. From their United Methodist tradition, the authors believe the authority of scripture to be informed by tradition, reason, and the bringing together of contextual experiences found in the diversity of the church. Their writing is influenced by a love for the church, and a desire to help hold together The United Methodist Church. Thus, they turn to the revelation science brings to an understanding of human sexuality, not to condemn those who find theological difference with the authors, but to commend all to common ground.

## Conclusion

United Methodists frequently return to the writings of their founder, John Wesley, for guidance when pondering moral issues. Wesley recognized and studied many of the issues of the Enlightenment which touched on Christianity. He valued new science and believed that science if understood could serve the cause of Christ and need not be feared. He was unwilling to subscribe to the antiscientific sentiments expressed by the leading Tory intellectuals of his day. Wesley pursued a middle road which considered both faith and reason. In assuming this position he offers a pattern for those, two centuries later, who seek to remain responsive to Christianity in a culture increasingly influenced by science. He summed up his thoughts about the role of science in *God's Approbation of His Works* (1782): “How small a part of this great work of God is man able to understand! But it is our duty to contemplate what He has wrought, and to understand as much of it as we are able.”

## Footnotes

1 Patients presented were observed by author BL and are typical of those described in the literature.
